# Cost-effectiveness and cost-utility evaluation of individual vs. group transdiagnostic psychological treatment for emotional disorders in primary care (PsicAP-Costs): a multicentre randomized controlled trial protocol

**DOI:** 10.1186/s12888-022-03726-4

**Published:** 2022-02-09

**Authors:** Ángel Aguilera-Martín, Mario Gálvez-Lara, Fátima Cuadrado, Eliana Moreno, Francisco García-Torres, José F. Venceslá, Jorge Corpas, Francisco J. Jurado-González, Roger Muñoz-Navarro, César González-Blanch, Paloma Ruiz-Rodríguez, Sara Barrio-Martínez, Maider Prieto-Vila, María Carpallo-González, Antonio Cano-Vindel, Juan A. Moriana

**Affiliations:** 1 Department of Psychology, Faculty of Education Sciences, University of Cordoba, C/ San Alberto Magno, s/n, 14071 Cordoba, Spain; 2 Maimónides Biomedical Research Institute of Cordoba, Reina Sofía University Hospital, C/ San Alberto Magno, s/n, 14071 Cordoba, Spain; 3 Department of Personality, Assessment and Psychological Treatments, Faculty of Psychology and Speech Therapy, University of Valencia, Av. Blasco Ibáñez, 21, 46010 Valencia, Spain; 4 Valdecilla Biomedical Research Institute (IDIVAL), Marqués de Valdecilla University Hospital, Av. Valdecilla, 25, 39008 Santander, Spain; 5 Castilla La Nueva Primary Care Centre, Health Service of Madrid, C/ Teruel, 4, 28941 Fuenlabrada, Spain; 6 Department of Experimental Psychology, Faculty of Psychology, Complutense University of Madrid, Campus de Somosaguas, s/n, 28223 Madrid, Spain

**Keywords:** Emotional disorders, Depression, Anxiety disorders, Somatoform disorders, Transdiagnostic therapy, Brief psychological treatments, Primary care, Randomized controlled trial, Cost-benefit analysis

## Abstract

**Background:**

Emotional disorders are common, and they have become more prevalent since the COVID-19 pandemic. Due to a high attendance burden at the specialized level, most emotional disorders in Spain are treated in primary care, where they are usually misdiagnosed and treated using psychotropic drugs. This contributes to perpetuate their illness and increase health care costs. Following the IAPT programme and the transdiagnostic approach, the PsicAP project developed a brief group transdiagnostic cognitive-behavioural therapy (tCBT) as a cost-effective alternative. However, it is not suitable for everyone; in some cases, one-on-one sessions may be more effective. The objective of the present study is to compare, in cost-benefit terms, group and individual tCBT with the treatment usually administered in Spanish primary care (TAU).

**Methods:**

A randomized, controlled, multicentre, and single-blinded trial will be performed. Adults with mild to moderate emotional disorders will be recruited and placed in one of three arms: group tCBT, individual tCBT, or TAU. Medical data and outcomes regarding emotional symptoms, disability, quality of life, and emotion regulation biases will be collected at baseline, immediately after treatment, and 6 and 12 months later. The data will be used to calculate incremental cost-effectiveness and cost-utility ratios.

**Discussion:**

This trial aims to contribute to clinical practice research. The involvement of psychologists in primary care and the implementation of a stepped-care model for mental disorders are recommended. Group therapy and a transdiagnostic approach may help optimize health system resources and unblock waiting lists so that people can spend less time experiencing mental health problems.

**Trial registration:**

ClinicalTrials.gov: NCT04847310; Protocols.io: bx2npqde. (April 19, 2021)

**Supplementary Information:**

The online version contains supplementary material available at 10.1186/s12888-022-03726-4.

## Background

Depressive, anxiety, and somatoform disorders are the most common mental health problems in the world [1, 2]. In 2019, the global prevalence of depressive disorders was 3.76 and 4.05% for anxiety disorders; Europe had the highest prevalence of the former (4.37%) and the second-highest of the latter (5.15%) [3]. The prevalence of both depressive (6.04%) and anxiety disorders (5.42%) in Spain is even higher; depression has continued to increase over the past few years to the point where the country has the third-highest prevalence after Greenland and Greece [3]. All these mental conditions have worsened during the COVID-19 pandemic [4, 5].

In Spain, mental health care is integrated in the national health system (at the specialized care level). It is free and unlimited because it is funded through public taxation. However, as in many other countries, users must have previously accessed primary care first. This is organized in territorially delimited community centres that employ multidisciplinary teams of general practitioners (GPs), nurses, and social workers but few psychologists (though some have been incorporated recently). Since specialized care waiting times are too long, many mental health cases are treated in primary care by GPs, who have consultations of less than 10 min and insufficient training in psychology to handle them [6]. This leads to misdiagnosis [7–9] and poor treatment (or even non-treatment) [6, 10], medication being the principal recourse. Spain is the eighth highest consumer of antidepressants of any OECD country, with 77 defined daily doses (DDDs) per 1000 habitants, which is double the OECD average [11]; and since 2010, anxiolytic and hypnotic use has increased by 200%, reaching in 91 DDDs in 2020 [12]. The use of psychotropic drugs as a first-line treatment may contribute to relapse and the duration of emotional disorders (EDs) [13] that generate large personal, social, and economic costs [14, 15]. Psychotherapy is the treatment of choice for common mental disorders due to its non-invasive nature [16]; patients tend to prefer psychological treatment, and though its short-term effects may be similar to those of pharmacotherapy, they can be more enduring [17–19]. Moreover, psychotherapy can save costs since it can be abbreviated when the patient or the context requires it, as happens in primary care [20].

According to Chisholm et al. [10], an increase in investment in care for depression and anxiety disorders would achieve very positive, long-term, cost-benefit ratios, substantially reducing the number of cases and increasing the number of healthy life-years. Some countries have begun to implement evidence-based psychological therapies in primary care settings. The Improving Access to Psychological Therapies (IAPT) programme [21] started in the UK in 2008. It provides community stepped-care low/high-intensity psychological treatment recommended by the National Institute for Health and Care Excellence (NICE) for EDs; cognitive-behavioural therapy (CBT) is the most widely used [16]. Through empirically-supported treatment and session-by-session monitoring, the IAPT programme has achieved great clinical and functional recovery rates with moderate to large effect sizes [21, 22]. It is now being replicated in other countries [23–25].

However, the IAPT programme has several limitations [21]. For example, whereas comorbidity is the most common clinical situation, its assessment and treatment processes are guided by categorical diagnoses, and this may affect therapy outcomes [26, 27]. Furthermore, some authors have observed that certain EDs improve with treatments that do not address them specifically [27]. As a result, a transdiagnostic approach has been developed that focuses on the dysfunctional emotion regulation strategies and cognitive processes that various mental disorders have in common [28–30]. In recent years, this approach has proven to be effective for reducing emotional symptoms and improving quality of life [31–33]. Moreover, transdiagnostic approaches might further reduce costs, as different conditions can be targeted in single group sessions.

Recently, the large national PsicAP study [34, 35] showed the effectiveness of a brief, group, transdiagnostic CBT compared with the treatment usually provided in Spanish primary care, achieving medium effect sizes in the reduction of emotional symptoms and recovery rates that were similar to those obtained by the IAPT programme. A recent trial by Corpas et al. [36] also obtained medium to high effect sizes for symptoms in the group intervention and very high effect sizes in the improvement of emotion regulation strategies and cognitive biases. However, some authors suggest that individual therapy may be more effective than group therapy, especially in the short-term [31, 37]. Some people prefer the former due to the fear of self-disclosure or the anxiety of social interaction [38]. In addition, the individual format also allows the therapist to establish a better relationship with the patient, which can benefit clinical outcomes [39]. The PsiBrief project [40, 41] compared brief versions of individual and group transdiagnostic CBT with the usual treatment in Spanish primary care. The results did not show any difference between the psychotherapy clusters in reducing emotional symptoms, though they were both more effective than primary care treatment (with moderate effect sizes). Unfortunately, the project did not report on patients’ treatment satisfaction or preference, and it is recommended that this should be considered when choosing the therapy [16]. Furthermore, as far as we know, few studies have included cost-benefit analyses of psychological treatments in primary care and even fewer transdiagnostic approaches.

### Objectives and hypotheses

We believe that the implementation of transdiagnostic therapy at the primary level using a stepped-care model would save costs and reduce waiting lists. Mild to moderate cases could be treated in primary care and severe ones could be referred to specialized care for combined and more intensive therapies.

The proposed trial aims to compare, in terms of cost-effectiveness and cost-utility, a brief, transdiagnostic CBT in two different formats, individual and group, with the treatment ordinarily administered in primary care (treatment as usual [TAU]) for mild to moderate EDs (i.e., depressive, anxiety, and somatoform disorders). We expect that:

#### Hypothesis 1

Individual treatment will be generally as effective as group treatment in reducing emotional symptoms and cognitive-emotional regulation biases (i.e., both will show similar post-intervention size effects).

#### Hypothesis 2

The TAU will be the least effective (i.e., with significantly lower post-intervention size effects).

#### Hypothesis 3

The group therapy will return the best results in terms of cost-effectiveness and cost-utility.

#### Hypothesis 4

The TAU will have the least cost-effectiveness and cost-utility ratios.

#### Hypothesis 5

The same results will be found across the follow-up assessments (6 and 12 months after intervention).

## Methods

This protocol has been registered at ClinicalTrials.gov (NCT04847310) and Protocols.io (bx2npqde) and follows the SPIRIT statement [42].

### Trial design

**Fig. 1:**
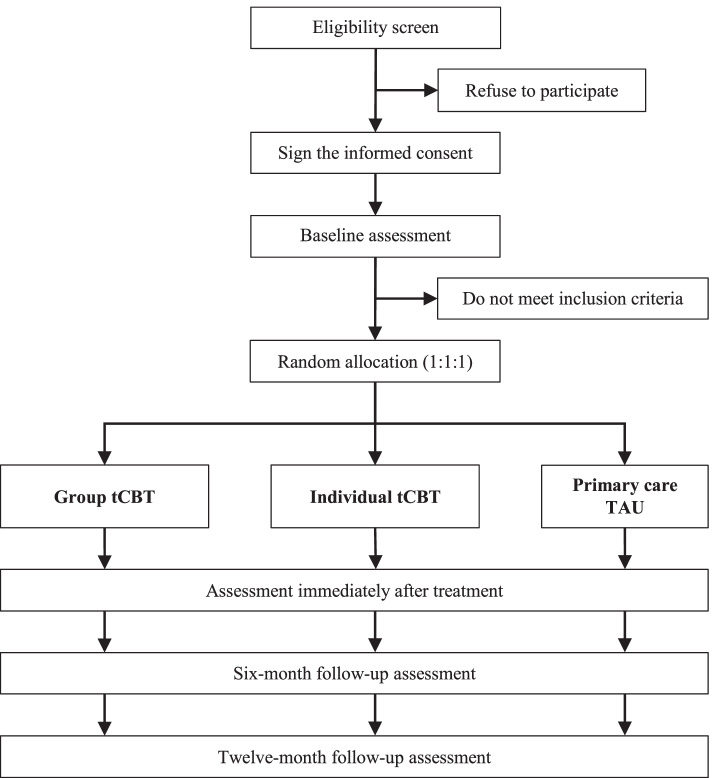
Study design flowchart. TAU: treatment as usual; tCBT: brief transdiagnostic cognitive-behavioural therapy

This intervention trial has a randomized, controlled, multicentre, and single-blinded design with 3 parallel groups, a 1:1:1 allocation ratio, and 4 measurement times: pre-intervention, immediately after the intervention, and two follow-ups (at 6 and 12 months; see Fig. 1).

### Study settings

The RCT will be performed in two primary care centres and one mental health unit in the province of Cordoba (region of Andalusia, Spain): the Carlos Castilla del Pino Health Centre, the Levante Sur Dr. Manuel Barragán Solís Health Centre, and the Community Mental Health Unit of Montilla.

### Eligibility criteria

Participants will be adult (18–65-year-old) men and women with mild to moderate EDs (i.e., depressive, anxiety, and/or somatoform disorders) according to the measurement tools (see Data Collection). People over 65 will be excluded to prevent outcomes from being distorted by age-related factors. Those who do not have an ED and/or have a severe mental illness (e.g., severe major depression, anxiety with alcohol abuse disorder, or comorbid psychotic symptoms); a history of frequent or recent suicide attempts; or a high level of functional impairment will also be excluded. People with severe somatoform symptoms will not be excluded unless they have a high level of disability. People who have difficulty understanding the Spanish language; have an intellectual or legal disability; are already participating in a clinical trial; or are undergoing private therapy sessions will be excluded. Potential participants will be asked about previous pharmacological treatment at pre-treatment to control that variable.

### Interventions

#### Group brief transdiagnostic cognitive-behavioural therapy (group tCBT)

It was developed by Cano-Vindel [43] and is based on the Unified Protocol for the Transdiagnostic Treatment of Emotional Disorders (UP) [44] and the IAPT programme [21]. It consists of seven 90-min sessions over 12–16 weeks and is conducted by a non-assessor clinical psychologist (hereafter the therapist), with 8–10 participants per group. Sessions are weekly or biweekly, and reduce in frequency as the intervention progresses; they are described below. (See Table 1 for the timeline.) The activities and homework are supported with materials such as theory documents, a CD for progressive muscle relaxation, self-recording sheets, and a therapy web. For more information, see González-Blanch et al. [43].

**Table 1:** Group tCBT’s session timeline [43]

Therapy contents	Sessions						
	1	2	3	4	5	6	7
1. Psychoeducation	**×**	**×**					
2. Relaxation	**×**	**×**					
3. Cognitive restructuring			**×**	**×**	**×**	**×**	**×**
4. Behavioural techniques				**×**	**×**	**×**	**×**
5. Relapse prevention							**×**

##### Session 1: introduction and psychoeducation (I)

The main objective of this session is to inform participants about the psychosocial nature of EDs and encourage them to play an active role in the treatment so that they attend all sessions and complete the assigned homework.

The first session begins with a presentation by the psychologist, who explains the protocol. The participants introduce themselves and talk about their symptoms and therapy expectations. They are informed about the group treatment: general objectives, components, functioning, timeline, and therapy rules (i.e., punctuality, confidentiality, respect, and notification if they are not going to attend), and provided with information about the treatment web [45] and its use. The psychoeducation then begins. They are given information about emotions, their adaptive role, and when they start to be maladaptive and become EDs. Participants are told how to manage stress and emotions cognitively, physiologically, and behaviourally, and how people sometimes magnify stimuli perceived as threatening or spend too much time thinking about a problem. (Both interpretational and attentional biases are therapy targets.) All this may be explained using personal examples drawn from the group members. Next, they are taught a diaphragmatic breathing technique, an emotion regulation tool to reduce psychophysiological activation. Finally, homework is given: participants are asked to read the therapy documents and online resources and practise the breathing technique with self-recording. The therapist gives homework to participants after each session and emphasizes the importance of completing it daily and putting the acquired knowledge into practice.

##### Session 2: psychoeducation (II) and relaxation

The objective of this session is to talk about the nervous system and the links between stress, coping, emotions, and health, as well as how to reduce psychophysiological activation through emotional self-regulation strategies.

This and the following sessions begin with a summary of the previous one and a review of the homework. The therapist discusses stress (i.e., definitions, theoretical models, phases, psychosomatic symptoms, and coping), the link between cognition and emotion, and presents strategies designed to downregulate the magnification and attention biases referred to in Session 1. Next, the diaphragmatic breathing technique is reviewed and practised, and progressive muscle relaxation is introduced as a useful emotional self-regulation strategy. The homework is given at the end: daily practice and self-recording of both diaphragmatic breathing and progressive muscle relaxation and the reading of an introductory document on cognitive therapy.

##### Session 3: cognitive restructuring (I)

The objective of this session is to introduce more emotional self-regulation strategies and teach participants to identify maladaptive thinking styles that generate and/or maintain emotional distress.

After the previous session summary and the review of the homework, participants talk about the usefulness of pleasurable activities and physical exercise. Then, the concept of irrational thinking (and how emotions can influence it) is introduced, and the integrative cognitive restructuring model [46] is explained. It consists of 3 phases: (1) information provided by the therapist regarding the problem (e.g., emotions, cognitions distorted, physiological responses, consequences, and emotional dysregulation problems); (2) self-observation, which implies the learning of complex cognitive concepts (e.g., cognitive biases, metacognition, and dysfunctional schemas), self-perception, and the recording thereof; and (3) cognitive restructuring, that is, training on the analysis and reassessment of threats, attention focus, problem-solving skills and how to improve self-efficacy and self-esteem. Some theoretical models are introduced for motivational purposes: the expectancy model and self-fulfilling prophecy (to illustrate cognition-behaviour interaction) [47] and the theory of attribution [48]. Finally, homework: the practice and self-recording of both relaxation techniques, pleasurable activities, physical exercise, self-observation (i.e., event, situation, cognition, physiological response, emotion, behaviour, and consequences), and the reading of a document on thinking distortions and cognitive biases.

##### Session 4: cognitive restructuring (II)

The objective of this session is to learn how to modify maladaptive thoughts through cognitive restructuring and positive self-instruction and to plan a behavioural experiment.

In this session, cognitive restructuring is practised using the homework self-observations, with an especial focus on the principal interpretational biases. Participants must (1) identify the cognitive processes that generate emotional distress; (2) identify the cognitive mistakes; and (3) turn them into more adaptive and rational thoughts. To encourage the latter, the positive self-instruction model [49] is introduced. The practice and self-recording of relaxation techniques, pleasurable activities, physical exercise, and cognitive restructuring are again given for homework; participants are also asked to design a personalized behavioural experiment to confront and disconfirm their irrational thoughts.

##### Sessions 5 and 6: cognitive restructuring (III) and problem-solving

These sessions continue with cognitive restructuring with positive self-instruction but behavioural training is now added. The therapist stresses the need to face stimuli that generate distress following successive approximation and reinforcement. This approach, coupled with cognitive restructuring, allows participants to disconfirm irrational beliefs and the anticipated negative consequences that encourage avoidance. The sessions also include several psychological techniques that are taught in the consultation for home practice: self-observation, stimulus control, reinforced behavioural training, exposure without security behaviours, and coping skills. A problem-solving technique [50] is introduced using an example from the group. These sessions are also used to reinforce group achievements and correct mistakes. The homework includes the practice and recording of all techniques learnt so far (i.e., diaphragmatic breathing, progressive muscle relaxation, pleasurable activities, physical exercise, cognitive restructuring, and behavioural experiments).

##### Session 7: relapse prevention and closing

The objective of this session is to review and reinforce the emotional self-regulation and cognitive-behavioural strategies learnt during the intervention. Finally, relapses are discussed not in terms of failure but challenges that have to be worked on, and the various techniques that have been learnt are generalized to other events and situations that may arise in the future.

#### Individual brief transdiagnostic cognitive-behavioural therapy (individual tCBT)

This is an adaption of the group therapy, with the same phases and the same order. However, since it is an individual intervention, its duration and the associated exercises can be personalized. It consists of a minimum of 6 and a maximum of 8 sessions of 30–60 min and is provided by a clinical psychologist not involved in the assessments.

#### Treatment as usual (TAU)

Participants in this group will be provided with common primary care treatment by a GP in a face-to-face consultation that seldom lasts more than 10 min. TAU usually consists of pharmacological treatment, though it might also involve practical advice or even no treatment at all [51]. The first consultation will count as part of the recruitment process and, if the patient agrees to participate in the trial, no therapy will be provided until they are allocated to a group. Once in the TAU intervention, if the practitioner recommends psychological treatment (e.g., referral to specialized care), the participant would be excluded to avoid contamination between clusters. TAU does not comprise a specific number of sessions; it will end when the GP considers the patient recovered.

### Therapist training

All the therapists are experienced clinical psychologists who work in the national health system. They study for 4–5 years to obtain a university degree and undergo a residency programme of 4 years to obtain clinical certification. They also undergo standardized training conducted by a supervisor PhD, when they learn the transdiagnostic therapy protocol. The course consists of studying a therapy handbook, four online lessons, and a face-to-face session with the trainer. All therapists will be supervised by a coordinator with whom they will be able to arrange follow-up sessions when they can resolve any doubts they may have during the intervention period.

### Outcomes

Primary outcomes are the screening of an ED and its severity. Secondary outcomes are the screening of other non-emotional mental disorders, symptom-related disability level, quality of life (general and health-related), the patient’s treatment satisfaction, and certain cognitive factors that have been observed to be common across different mental disorders [28–30]. Changes over time in all variables will be analysed (from pre-treatment to post-treatment and follow-ups) to assess treatment effectiveness. In addition, sociodemographic and medical data will be collected. Medical data will be used to calculate health care costs for cost-effectiveness analyses. Finally, health-related quality of life will be used to calculate the quality-adjusted life years (QALYs) for cost-utility analyses.

### Timeline

Table 2 shows the timeline as recommended in the SPIRIT statement [42].

**Table 2:**
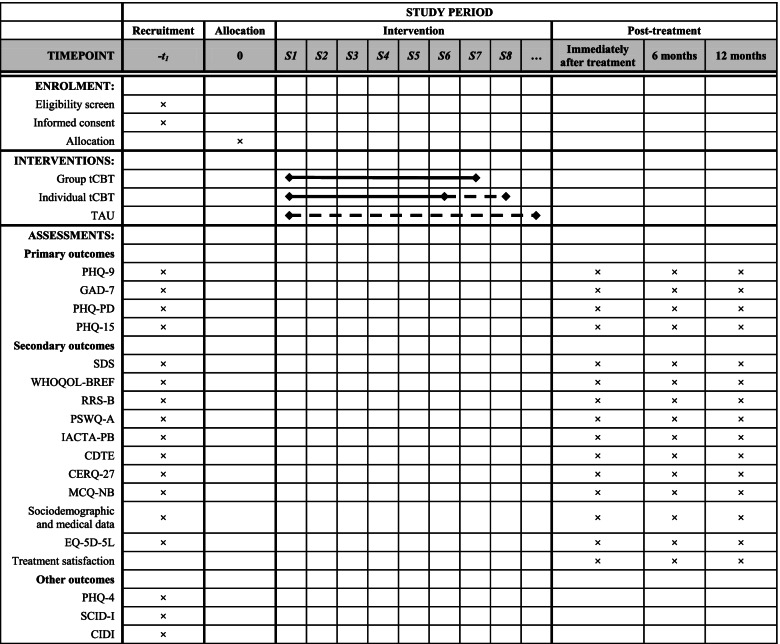
Study timeline according to SPIRIT Statement [42]

*CDTE* Questionnaire of Cognitive Distortions in Emotional Disorders, *CERQ* Cognitive Emotion Regulation Questionnaire, *CIDI* Composite International Diagnostic Interview, *EQ-5D-5L* 5-dimension, 5-level European Quality of Life scale, *GAD* Generalized Anxiety Disorder, *IACTA-PB* Inventory of Cognitive Activity in Anxiety Disorders-Panic Brief subscale, *MCQ-NB* Metacognitions Questionnaire-Negative Beliefs subscale, *PHQ* Patient Health Questionnaire, *PSWQ-A* Penn State Worry Questionnaire-Abbreviated, *RRS-B* Ruminative Responses Scale-Brooding subscale, *SCID-I* Structured Clinical Interview for DSM Axis-I Disorders, *SDS* Sheehan Disability Scale, *TAU* Treatment as usual, *tCBT* Transdiagnostic cognitive-behavioural therapy, *WHOQOL-BREF* WHO Quality of Life scale-Brief version

### Sample size

We used effect sizes from the previous literature to determine the sample size. Corpas et al.’s primary care RCT [41] compared both group and individual brief tCBT with TAU for emotional disorders and found low to medium effect sizes for all outcomes. Individual effects were larger (Cohen’s *d* ranged from .54 to .74) than group effects (.4 to .63) when compared with TAU; however, there was no significant difference between both transdiagnostic formats, with (very) low effect sizes (.06 to .27). Another primary RCT by Corpas et al. [36] compared tCBT with TAU and found medium to large effect sizes (from .39 for depression to .81 for panic symptoms). Finally, the PsicAP study compared the group tCBT used herein to TAU in primary care. Intention-to-treat analyses showed low to medium effect sizes over time for depression (Morris’ *d* ranged from .36 to .58), anxiety (.38 to .65), and somatizations (.31 to .4). Subsequently, we decided to assume a medium effect size of .6 (Cohen’s *d*).

Since software to determine sample sizes for linear mixed model analyses was not available, we used the *f* index of G*Power [52]. We assumed an effect size of .3 (the equivalent *f* value); a statistical power (1 - *β*) of .8; and a significance level (*α*) of .05, obtaining a required sample size of 111. However, to control withdrawals and take into account the abandonment rates in other similar trials [36, 41], we assumed a dropout rate of 15%, which brought the required sample size to 128.

### Recruitment

Participants will be recruited from primary care by their GPs. All patients aged 18–65 who are considered to meet eligibility criteria (based on their previous history of ED, diagnostic criteria, or clinician suspicion) will be encouraged to participate. After signing an informed consent form, the participant will have an appointment with an assessor psychologist, who will administer the measurement tools. If the participant does not meet the criteria, they will be referred back to their GP. If the participant is excluded due to a severe mental illness or a high-level disability, the GP will be advised to refer them to specialized care. Patients will receive no economic compensation for their participation in the trial.

### Allocation

The sequence will be generated before recruitment and allocation will be accomplished by a non-assessor investigator using computer software that randomly assigns participants to the three parallel clusters with a 1:1:1 ratio. Participants will receive the allocation information via email.

### Blinding

The trial will be single-blinded. Assessors and data managers will be blinded insofar as they will have no knowledge of the treatment allocation and will not be involved in the interventions. However, even though participants will be blinded during the pre-treatment evaluation, they will probably not remain blinded once allocated, since the experimental conditions are qualitatively different. For the same reason, the clinical psychologists who perform the transdiagnostic treatments will not be blinded. Since participants might consult their GPs during the intervention period, they will be asked not to share information concerning their allocation; this will ensure that the GPs are blinded.

### Data collection

#### Primary outcomes

Data regarding mental disorders will be collected through the Patient Health Questionnaire (PHQ) [53], a self-reported version of the PRIME-MD. We will use the subscales of the Díez-Quevedo et al.’s Spanish validated version [54] (except where indicated) to recruit participants and determine the severity of their emotional symptoms.

##### Depression symptoms

Participants will be evaluated using the 9-item PHQ subscale (PHQ-9) [55], which scores the 9 DSM-IV depression criteria present in the previous 2 weeks from 0 (*not at all*) to 3 (*nearly every day*). A score of 10 (at least 5 items rated with 2 [Item 9 also scores 1], and with Item 1 or 2 amongst them) is usually the cut-off point for major depression disorder (MDD): a score of 10–14 indicates minor depression, moderate MDD, or dysthymia; 15–19, moderately severe MDD; and 20–27, severe MDD. This tool has been tested in Spanish primary care centres (McDonald’s *ω* = .89) [56], when 10 was validated as the cut-off point for MDD screening (a sensitivity of 95% and a specificity of 67%).^1^

##### Anxiety symptoms

The 7-item Generalized Anxiety Disorder scale (GAD-7) [57] assesses common anxiety symptoms for the previous 2 weeks from 0 (*not at all*) to 3 (*nearly every day*). Cut points of 5, 10, and 15 represent mild, moderate, and severe anxiety, respectively. The algorithm sets 8 as the cut-off point for GAD, though it has been found that a score of 10 is more optimal [57]. We will use the version validated by García-Campayo et al. [58]. This was tested recently in primary care centres (Cronbach’s *α* = .83) [59], when 10 was confirmed as the best GAD screening criterion (a sensitivity of 87% and a specificity of 78%).

##### Panic symptoms

Since panic attacks can appear with any mental disorder (not just anxiety) [60], these symptoms should be measured separately. The Panic Disorder PHQ subscale (PHQ-PD) [61] is the specific panic disorder module of the PHQ and scores each DSM-IV criterion as *yes* or *no*. Muñoz-Navarro et al. [62] tested it in Spanish primary care settings and modified the original algorithm to increase the sensitivity for PD diagnosis: the most optimal cut-off point for screening purposes was 5 (the first item [for panic screening] and one of the following 3, plus 4 somatic symptoms; a sensitivity of 77% and a specificity of 72%).

##### Somatization

The 15-item PHQ subscale (PHQ-15) [63] is the somatization module of the PHQ. It scores symptoms present in the previous 4 weeks from 0 *(not bothered at all*) to 2 (*bothered a lot*). It includes 13 somatic symptoms plus 2 from the PHQ-9 (sleeping problems and fatigue); cut points 5, 10, and 15 represent low, medium, and high somatic symptom severity, respectively. However, whereas the original algorithm requires a score of at least 2 in 3 or more somatic symptoms to screen a somatization disorder (a sensitivity of 78% and a specificity of 71%) [64], the cut-off point usually used is 10, as it is in the PHQ-9 and the GAD-7 [65]; nevertheless, since this number can be obtained with 10 symptoms rated with the minimum severity, we decided to join both criteria. Furthermore, although an absence of biological cause is also often required (since PHQ-15 does not distinguish between medically explained and unexplained symptoms) [63], the self-administered nature of the PHQ-15 makes it difficult for the subject to determine this. This tool has been validated with Spanish psychiatric outpatients (*α* = .78) [66].

#### Secondary outcomes

##### Eating disorders and alcohol abuse

The PHQ can also be used to measure the presence of other mental disorders. The Spanish version [54] detects eating disorders such as bulimia nervosa or binge eating disorder (a sensitivity of 92% and a specificity of 98%) and alcohol abuse (a sensitivity of 76% and a specificity of 99%). Affirmative answers to Items 6a to 6c and 8 indicate bulimia nervosa; a negative answer (or no answer at all) for Item 8 points to a binge eating disorder. An affirmative answer to either Item 10a to 10e indicates alcohol abuse. In any of these cases, the patient would be interviewed by a clinical psychologist to confirm a possible diagnosis of eating, alcohol, or personality disorder.

##### Level of impairment

The Sheehan Disability Scale (SDS) [67] is a self-administered test that measures subjective symptom-related impairment with 5 11-point Likert items. The first 3 items rate key areas in the previous month: work, social life/leisure activities, and family life/home responsibilities. Two additional items assess stress level and perceived social support in the previous week. We will use the Spanish version developed by Bobes et al. [68] because it has shown good properties in primary care populations (*α* = .83) [69]. One, 4, and 7 are the cut points for mild, moderate, and high disability, respectively.

##### General quality of life

Psychological, physical, social, and environmental domains will be assessed through the 26 5-point Likert items of the World Health Organization Quality of Life Instrument-Brief (WHOQOL-BREF) [70], the abbreviated version of the 100-item WHOQOL (WHOQOL-100) [71]. The more the participant scores, the better quality of life. The WHOQOL has been validated in Spanish populations [72, 73] and has shown good psychometric properties (*α* > .7) in psychological, physical, and environmental domains, though its internal consistency in social domains has varied from .58 [73] to .75 [72].

##### Ruminative thinking

The 22-item Ruminative Responses Scale (RRS) [74] was originally developed to measure ruminative responses to depressed mood. It has been validated in a Spanish population [75]; however, only the 5-item brooding factor subscale (RRS-B) will be used in the proposed trial (*α* = .79) [76]. The RRS-B scores how often the participant thinks as described in each item (1 = *almost never* to 4 = *almost always*) when they are discouraged, sad, or depressed.

##### Worry

The Penn State Worry Questionnaire (PSWQ) [77] measures pathological worry as an uncontrollable and general state (i.e., as a GAD feature). It has been validated in Spain [78]. The proposed study will use an 8-item abbreviated version (PSWQ-A) [79] that has already shown good properties in primary care (*α* = .9) [76]. The PSWQ-A items rate how far worries affect the person (1 = *not at all typical of me* to 5 = *very typical of me*).

##### Attentional and interpretational biases

The Inventory of Cognitive Activity in Anxiety Disorders (IACTA) was originally developed by Cano-Vindel [80]. It includes several subscales that assess distortions according to Eysenck’s four-factor theory [81]. The 5-item, abbreviated panic version, the IACTA-Panic Brief (IACTA-PB; *α* = .87) [76] will be used to measure attentional biases. It scores how often the participant has certain cognitive distortions (0 = *almost never* to 4 = *almost always*). In addition, we will use the Questionnaire of Cognitive Distortions in Emotional Disorders (CDTE) [The PsicAP Group: Cuestionario de Distorsiones Cognitivas en Trastornos Emocionales, unpublished], an under-review tool that scores the frequency of certain cognitive biases in the main EDs (i.e., MDD, GAD, PD, and somatization disorder) from 0 (*almost never*) to 4 (*almost always*). It includes 16 items that measure the presence of four factors: sustained attention bias (*α* = .96); divided attention bias (*α* = .95); magnification interpretational bias (*α* = .94); and catastrophization interpretational bias (*α* = .96), with high levels of discriminant validity amongst the four EDs (ROC values > .8).

##### Emotion regulation strategies

The 36-item Cognitive Emotion Regulation Questionnaire (CERQ-36) [82] measures the specific cognitive emotion regulation strategies a person uses to face a stressful event. It scores how often the participant thinks as described (1 = *almost never* to 5 = almost always). The CERQ has been validated in a Spanish population [83]; the 18-item shortened version [84] will be used in the proposed study (*α* values range from .84 [*adaptive*] to .72 [*less adaptive*]).

##### Metacognitions

The 30-item Metacognitions Questionnaire (MCQ-30) [85] is a short version of the original MCQ [86], which measures beliefs about one’s own thinking processes. It has been validated in a Spanish population [87]. In the proposed trial, the 6-item *negative beliefs* (concerning uncontrollability/danger) subscale (MCQ-NB; *α* = .82) will be used [76]. This scores how far the patient agrees with the sentences presented to them (1 = *totally disagree* to 4 = *totally agree*).

##### Participant data and treatment satisfaction

An ad hoc questionnaire will be used to collect socio-demographic (gender, age, civil status, educative level, employment situation, and income level) and ED-related medical data (public and private health care consultations, accidents, medical tests, and sick leave in the previous 3 months, psychotropic drugs or other medications, and their posology). It includes an additional question about treatment satisfaction (at post-treatment and follow-ups). Medical records will be also consulted (though, for privacy reasons, only strictly necessary data will be collected).

##### Cost and utility data

The medical data collected above will also be used for cost calculations. In addition, cost-utility will be measured through the European Quality of Life Scale (EuroQoL, EQ) [88]. The Spanish version of the 5-domain, 5-level EuroQol (EQ-5D-5L) [89, 90] will be used to calculate the QALYs. The EQ-5D-5L measures 5 domains of health-related quality of life (mobility, self-care, daily activities, pain/unease, and anxiety/depression) through 5 severity levels (*no problems*, *slight problems*, *moderate problems*, *severe problems*, and *extreme problems*). This makes it possible to establish up to 3125 different health states, each of which can be represented through an index value that reflects the health state quality contextualized in the person’s country/region. It also includes a visual analogue scale (VAS) that scores the respondent’s current subjective, general health state from 0 to 100. For more information, see van Reenen et al. [90].

#### Collection process

First, the 4-item PHQ ultra-brief subscale (PHQ-4) [91] will be used for the recruitment phase. The PHQ-4 gathers 2 items from the PHQ-2 and 2 from the GAD-2 (short versions of the PHQ-9 and GAD-7, respectively). It has been used in a Spanish primary care population (Spearman-Brown’s *ρ*_PHQ-4_ = .72; *ρ*_PHQ-2_ = .86; *ρ*_GAD-2_ = .76) [92]. A score greater than or equal to 3 would indicate the need for additional assessment (PHQ-2: a sensitivity of 90% and a specificity of 61%; GAD-2: a sensitivity of 88% and a specificity of 61%). It can be an extremely useful tool, as it helps to accelerate the screening process. It has been suggested, however, that both PHQ-2 and GAD-2 sum scores should be regarded separately in primary care samples [92]. The first item from the PHQ-PD has been added to screen panic disorder.

Second, depression, anxiety, panic, and somatizations will be assessed through the PHQ subscales mentioned above. If a participant scores as having a severe ED (depressive or anxiety disorder) or a non-emotional mental illness, or if the diagnosis is not clear, they will undergo a second evaluation with a gold-standard tool. The Structured Clinical Interview for DSM Axis-I Disorders (SCID-I) [93] will be used to assess panic and depression disorders and the Composite International Diagnostic Interview (CIDI) [94] will be used for GAD (since the former may not be adequate as it only includes one item for GAD). If these tools confirm the PHQ results, the patient will be referred to their GP, who will refer them to specialized care. The same will apply to those who score high in the SDS. All measures, except the PHQ-4, the SCID-I, the CIDI, and the treatment satisfaction question, will be collected at baseline, immediately after treatment, and 6 and 12 months later (see Table 2). Data will be also collected from medical records for the 3 months before participation in the study. To facilitate completion, the questionnaires may be answered in person, by email, online, or by phone.

To reduce the number of withdrawals, the clinical psychologists will telephone the participants to encourage them to continue with the treatment and/or to participate in the follow-up assessments. People who drop out of the intervention will still be invited to complete the questionnaires, especially at the first post-treatment assessment (immediately after the intervention).

### Data management

Scores from both electronic and paper questionnaires will be tabulated in SPSS Statistics. Data from the online instruments will be exported to SPSS.

### Statistical methods

#### Analysis of clinical effectiveness

Data analysis will be carried out using SPSS Statistics. Effectiveness-related data will be analysed using intention-to-treat and per-protocol approaches. After the homogeneity of intra- and inter-groups is checked, changes over time (baseline, post-intervention, and follow-ups) in primary and secondary outcomes will be analysed using linear mixed models (LMMs), since this method has been recommended rather than ANOVA or MANOVA analyses because LMMs do not require participants with missing values to be omitted nor imputed, and they are more adequate for repeated measures [95]. Likewise, effect sizes (Cohen’s *d* with bias corrections) will be calculated, as well as their accuracy (by taking into account the number of treatment sessions). The percentage of patients in each cluster who experience a 50% decrease in the number of clinical symptoms and scores to one standard deviation and the percentage of cases with a probable ED before and after receiving treatment (according to cut-off criteria) will be calculated. Therapeutic success criteria will be determined by obtaining post-intervention means significantly lower (*p* ≤ .05) and medium/large effect sizes significantly higher than those of the controls, especially in the ED scores. Clusters will be also compared regarding impairment, quality of life, emotion regulation biases, and satisfaction with treatment.

#### Cost analysis

Cost-related data will be collected through medical records and ad hoc questionnaires, from 3 months before inclusion in the study to 12 months after the intervention. Direct costs will be calculated by adding the ED-related costs due to medication use (antidepressants, anxiolytics, hypnotics, and sedatives), medical tests and other health services, and health personnel (primary/specialized care and public/private care). Medication costs will be calculated by multiplying price per milligram (€/mg) according to the Vademecum International (including VAT) [96] by the daily dose (mg) and the number of days of drug treatment. Cost data relating to medical tests and the use of health services will be obtained through the fee information published on the Andalusian Health Service’s official website [97]. Since a group psychotherapy session does not have a specific tariff, it will be considered as a GP consultation without medical tests because GPs and clinical psychologists have similar basic salaries [98]. Indirect costs will be calculated by multiplying the days of ED-related sick leave by the participant’s current daily minimum salary; the expense of replacement workers will also be factored in when incurred. Total costs will be obtained by summing direct and indirect costs.

#### Analyses of cost-effectiveness and cost-utility

Cost-effectiveness analysis will be conducted by calculating incremental cost-effectiveness ratios (ICERs), which are defined as the difference in mean costs between interventions divided by the difference in their effectiveness according to the participants’ mean scores (i.e., one ICER per each comparison between two clusters and per each instrument). However, cost-effectiveness analyses are open to question since they rate the more appropriate intervention based only on the clinical perspective. Cost-utility analyses use the intervention health-related utilities, subjectively rated by participants. Therefore, they depend on a social perspective, that is, the participants express their preferences based on the value they assign to their health status. The EQ-5D-5L will be used to calculate those utilities as QALYs, and the latter will be used to obtain the incremental cost-utility ratios (ICURs), defined as the difference in mean costs divided by the difference in mean QALYs (i.e., one ICUR per each comparison between two clusters). Since follow-ups will not go beyond 12 months post-intervention, neither costs nor results will be subject to discount. The bootstrapping method (a resampling method) will be used to obtain more accurate ICERs and ICURs. Missing data will be analysed through Student’s *t* and *χ*^2^ tests for ED severity level, sex, and age; this will allow us to know whether missing data due to dropout are related to chance. Finally, a sensitivity analysis will be carried out to test the robustness of cost-effectiveness and cost-utility results.

### Monitoring

The proposed trial has no data monitoring committee since the potential harms are limited to the pharmacological treatment that is ordinarily provided in primary care. Study progress will be supervised through regular contacts and meetings between the intervention professionals and the principal investigator. All updates will be published through the online registers: ClinicalTrials.gov (NCT04847310) and Protocols.io (bx2npqde).

## Discussion

This protocol tries to combine the strengths of the PsiBrief and the PsicAP projects. The large PsicAP project [34, 35] studied a brief group transdiagnostic intervention as a more cost-effective possibility for ED treatment in primary care; however, as has been noted, group therapy is less flexible (and less popular amongst patients) than individual therapy (which allows for stronger therapeutic relationships). Meanwhile, the PsiBrief project [40, 41], which introduced individual therapy, saw a high attrition rate, so follow-ups were not possible. Also, it did not include cost-benefit analyses. The proposed study will try to combine the best of each of these projects, comparing the cost-effectiveness and cost-utility of both group and individual transdiagnostic therapy with TAU in primary care.

Based on PsiBrief and PsicAP’s results, we hypothesize that our proposed experimental interventions will be equally effective in reducing emotional symptoms and improving emotion regulation strategies, and TAU will be the least effective. Since group therapy involves several patients with different symptoms, we hypothesize that it will have the best outcomes in terms of cost-effectiveness and cost-utility, and TAU the worst. Finally, we hypothesized that these results will be sustained in the follow-ups.

### Anticipated limitations

We recognize that our protocol has several limitations, and we expect difficulties in its execution. First, the trial design excludes some groups to minimize confusion variables (minors, older people, and patients with severe mental disorders and/or with intellectual, legal, or high functional disability), so additional research will have to be carried out on these populations; and the single-blind approach will not allow us to control patients’ therapy expectations, though participant blinding is ordinarily not feasible in RCTs that compare psychotherapies.

Second, we might encounter obstacles during recruitment. In the first phase, the great care saturation faced by GPs (i.e., they have to deal with great numbers of patients in a short time) might make enrolment difficult; moreover, since there has never been a psychotherapy culture in primary care, some of them might have little motivation to collaborate. In the second phase, the main limitation might be the measurement tools. Brief instruments have been included in the protocol because primary care requires rapid assessment; however, they do not have the same diagnostic accuracy as gold-standard tools such as the SCID. Furthermore, because these questionnaires are self-administered, completion and the veracity of answers are less controlled than clinical interviews. Nonetheless, the tools included have been validated and tested in Spanish primary care, and they accelerate the diagnostic process in clinical practice.

Third, the interventions might have limitations. For example, each treatment may have a different duration, especially TAU, so this might impact effectiveness (though we will consider this in our analyses). Also, each intervention will be carried by different personnel (i.e., GPs and clinical psychologists), so this might affect the results. In addition, TAU might generate expectations in participants, and they cannot be feasibly blinded. An alternative may be sham therapy, but TAU is closer to clinical reality, so it provides better external validity. The level of adherence to the protocol is another difficulty envisaged, though the therapists will be supervised by PhD trainers during the treatment period.

Finally, this kind of trial risks a high dropout rate and missing data, especially at follow-ups. Unlike the IAPT programme, the proposed study will not take session-by-session measurements, because the number of instruments we are using would make it difficult. We hope, therefore, that the clinical psychologists will encourage participants to complete the measurements through regular telephone calls. Finally, due to the duration of the study, intervention and measurements might be affected by patients’ availability (holidays, medical appointments, and so on), but we will make an effort to ensure that timings are similar between groups.

### Future directions

If our results show the economic feasibility of including psychotherapy in primary care, they may help to change health care policies and implement empirically-supported psychological treatments following a stepped-care model. Patients with mild to moderate conditions would be treated in primary care and those with severe mental disorders would be referred to specialized care involving more intensive therapies that combine psychological and pharmacological approaches. As a result, primary care resources would be optimized, waiting lists would be unblocked, and patients would spend less time of their lives with their disability. Once such a model is implemented, the next step might be the development of internet-based psychotherapies through the use of websites or apps, which may be more cost-effective and accessible for some people.

However, even though empirically-supported psychotherapies may improve patients’ symptoms and quality of life, they cannot reduce prevalence rates; only prevention can do that. Investment is needed both in psychological treatment and prevention. If we can resolve people’s mental problems before they become disorders, we might save not only costs but also a great deal of suffering.

#### Protocol status

This project started in September 2021.

## Supplementary Information

**Additional file 1.**

## Data Availability

Datasets generated and analysed during the proposed study will be available from the corresponding author on reasonable request.

## Abbreviations

ED: Emotional disorder
CBT: Cognitive-behavioural therapy
CDTE: Questionnaire of Cognitive Distortions in Emotional Disorders
CERQ: Cognitive Emotion Regulation Questionnaire
CIDI: Composite International Diagnostic Interview
DDD: Defined daily doses (per 1000 habitants)
DSM: Diagnostic and Statistical Manual of Mental Disorders
EQ-5D-5L: 5-domain, 5-level European Quality of Life Scale
GAD: Generalized anxiety disorder
GP: General practitioner
IACTA-PB: Inventory of Cognitive Activity in Anxiety Disorders-Panic Brief version
IAPT: Improving Access to Psychological Therapies
ICD: International Classification of Diseases
ICER: Incremental cost-effectiveness ratio
ICUR: Incremental cost-utility ratio
LMM: Linear mixed models
MCQ-NB: Metacognitions Questionnaire-Negative Beliefs subscale
MDD: Major depression disorder
NICE: National Institute for Health and Care Excellence
OECD: Organisation for Economic Co-operation and Development
PD: Panic disorder
PHQ: Patient Health Questionnaire
PSWQ-A: Penn State Worry Questionnaire-Abbreviated
QALY: Quality-adjusted life year
RCT: Randomized controlled trial
RRS-B: Ruminative Responses Scale-Brooding subscale
SCID: Structured Clinical Interview for DSM
SDS: Sheehan Disability Scale
TAU: Treatment as usual
tCBT: (brief) transdiagnostic cognitive-behavioural therapy
UP: Unified Protocol for the Transdiagnostic Treatment of Emotional Disorders
WHOQOL: World Health Organization Quality of Life Instrument

## Footnotes

1 Twelve was found as the most appropriate cut-off point in Spanish primary care population [56]; however, since this is based on a single study, we decided to use 10 as it is the most used cut-off point.
