# Supplementary material for: Cost-effectiveness and cost-utility evaluation of individual vs. group transdiagnostic psychological treatment for emotional disorders in primary care (PsicAP-Costs): a multicentre randomized controlled trial protocol
Source: BMC Psychiatry. 2022 Feb 9;22:99. doi: 10.1186/s12888-022-03726-4 (PMC8826705; doi:10.1186/s12888-022-03726-4)
Supplement: Supplementary file 1 — Additional file 1. [file 12888_2022_3726_MOESM1_ESM.docx]

**Appendix 1.** WHO trial registration data set.

| **Data category** | **Information** |
| --- | --- |
| Primary registry and trial identifying number | ClinicalTrials.gov  NCT04847310 |
| Date of registration in primary registry | April 19, 2021 |
| Secondary identifying numbers | Protocols.io  bx2npqde |
| Source(s) of monetary or material support | Ministerio de Ciencia, Innovación y Universidades (Gobierno de España) [Ministry of Science, Innovation and Universities (Government of Spain)] |
| Primary sponsor | Universidad de Córdoba [University of Cordoba] |
| Secondary sponsor(s) | Agencia Estatal de Investigación [State Investigation Agency] |
| Contact for public queries | Juan Antonio Moriana, PhD  +34 957212093  ed1moelj@uco.es |
| Contact for scientific queries | Juan Antonio Moriana, PhD  University of Cordoba  Cordoba, Spain |
| Public title | Cost-Effectiveness and Cost-Utility Evaluation of the Individual vs. Group Transdiagnostic Psychological Treatment for Emotional Disorders in Primary Care (PsicAP-Costs): A Multicentre Randomized Controlled Trial Protocol |
| Scientific title | Cost-Effectiveness and Cost-Utility Evaluation of the Individual vs. Group Transdiagnostic Psychological Treatment for Emotional Disorders in Primary Care (PsicAP-Costs): A Multicentre Randomized Controlled Trial Protocol |
| Countries of recruitment | Spain |
| Health condition(s) or problem(s) studied | Emotional disorders (depression, anxiety, and somatoform disorders) |
| Intervention(s) | Experimental conditions: (1) group, brief, transdiagnostic, cognitive-behavioural therapy; (2) individual, brief, transdiagnostic, cognitive-behavioural therapy  Control condition: treatment as usual (usually pharmacological) |
| Key inclusion and exclusion criteria | Age: 18-65  Gender: both  Accepts healthy volunteers: no  Inclusion criteria: mild to moderate emotional disorders  Exclusion criteria: no emotional disorder, severe mental illness, high disability level, recent severe suicide attempt |
| Study type | Interventional  Allocation: randomized  Intervention model: parallel assignment  Masking: single blind (assessors)  Design: multicentre controlled trial with pre-post measures and follow-ups  Primary purpose: treatment |
| Date of first enrolment | September 2021 |
| Target sample size | 128 |
| Recruitment status | Ongoing |
| Primary outcome(s) | Changes in depression, anxiety, and somatization symptoms [Baseline, immediately after treatment, and 6 and 12 months later] |
| Key secondary outcomes | Changes in subjective disability, quality of life (general and health-related), ruminative thinking, pathological worry, attentional and interpretational biases, cognitive emotion strategies, and metacognitions [Baseline, immediately after treatment, and 6 and 12 months later]  Changes in treatment satisfaction [Immediately after treatment and 6 and 12 months later]  Medical data (consultations, medical tests, medication, etc.) [Baseline, immediately after treatment, and 6 and 12 months later] |
